# Identification of miRNAs Involved in Lipid Metabolism and Tuber Development in *Cyperus esculentus* L.

**DOI:** 10.3390/plants13233305

**Published:** 2024-11-25

**Authors:** Yunfei Gao, Le Wang, Shanshan Cao, Liangyu Chen, Xueying Li, Weixuan Cong, Songnan Yang, Jian Zhang, Xiaojun Nie, Jun Zhang

**Affiliations:** 1 College of Agronomy, Jilin Agricultural University, Changchun 130118, China; 20210055@mails.jlau.edu.cn (Y.G.); zhangjian@jlau.edu.cn (J.Z.); 2 College of Plant Science, Jilin University, Changchun 130062, China; lewang27@jlu.edu.cn; 3 Department of Biology, University of British Columbia, Okanagan, Kelowna, BC V1V 1V7, Canada; 4 State Key Laboratory for Crop Stress Resistance and High-Efficiency Production, College of Agronomy, Northwest A&F University, Xianyang 712100, China; 5 National Crop Variety Approval and Characteristic Identification Station, Jilin Agricultural University, Changchun 130118, China

**Keywords:** *Cyperus esculentus*, miRNAs, GC-MS, oil synthesis, tuber development

## Abstract

Tiger nut (*Cyperus esculentus* L.) is recognized for its high oil and oleic acid content in underground tubers. However, the molecular mechanisms governing growth, development, and fatty acid accumulation in these tubers are not well understood. This study employed gas chromatography–mass spectrometry (GC–MS) and small RNA sequencing on tiger nut tubers across five developmental stages. The findings indicate that the critical period for accumulating dry matter and oils, particularly oleic acid, occurs between 35 and 75 days after tuber formation. A total of 183 microRNAs (miRNAs) were identified, comprising 31 known and 152 novel miRNAs. Approximately half of these miRNAs (such as ces-miR156b-3p and ces-miR166a-3p) exhibited differential expression during and around the key periods of metabolite synthesis. The predicted target genes of these miRNAs were significantly enriched in glycerate 3-phosphate metabolism and cell growth processes. Furthermore, 13 miRNA–mRNA interaction modules related to oil accumulation and tuber growth were identified, and these target genes’ expression levels showed significant differences during the tuber developmental stages. These findings advance the understanding of the molecular mechanisms underlying tuber development and oil accumulation in tiger nut.

## 1. Introduction

Tiger nut (*Cyperus esculentus* L.), a C4 plant in the *Cyperaceae* family, primarily yields tubers as its main agricultural product [1,2]. These tubers accumulate substantial amounts of starch (23.2–29.9%) and oil (20.1–34.5%), and contain bioactive compounds including organic acids, alkaloids, and phenolics [3,4]. The tuber oil is predominantly composed of monounsaturated fatty acids, such as oleic acid, which impart exceptional oxidative stability and physicochemical properties [5,6]. This composition makes it suitable as a healthy edible oil and a renewable raw material for biodiesel and other lipid-based chemical industries [7]. The estimated tuber yield of tiger nut can reach up to 12 t/ha, which can produce an oil output of 1.5 t/ha, significantly surpassing conventional oil crops like soybean and peanut [5]. Additionally, tiger nut demonstrates excellent soil adaptability, strong resistance to poor soil conditions, low input costs, and resilience to pests and diseases [8]. Therefore, research on the development of tiger nut tubers and oil synthesis mechanisms will not only aid its genetic improvement but also elucidate the molecular mechanisms of oil production in plants.

Research indicates that tiger nut tubers exhibit rapid exponential growth in their early stages, transitioning to metabolite accumulation once they reach a certain size [9]. While the timing of new tuber formation varies after sowing due to the different cultivars, the phase of rapid oil accumulation typically occurs 20 to 70 days post-formation [6,9,10]. There is a significant accumulation of lipid droplets during tuber development, with lipid composition analysis identifying triacylglycerol (TAG) as the primary contributor to the increased tuber oil content [6]. Transcriptomes across various developmental stages reveal that differentially expressed genes in the early stages are highly enriched in glycerolipid and linoleic acid synthesis pathways [6]. Key candidate enzymes involved in the oil synthesis of encoding genes, such as acyl-ACP thioesterase (FATA) and diacylglycerol acyltransferase (DGAT), are activated early and sustain high expression levels until tuber maturation. In contrast, stearoyl–ACP desaturase (SAD) and phosphatidate phosphohydrolase (PAH) are activated and show expression during the mid- to late developmental stages. Comparative transcriptomic studies with the related species *Cyperus rotundus* have revealed that the expression levels of candidate genes involved in oil synthesis are significantly higher in tiger nut; for example, *FATA* is 10.3 times higher [8]. Furthermore, the transcription factors ABSCISIC ACID INSENSITIVE3 and WRINKLED 1 are crucial in regulating the expression of these oil synthesis-related genes. In addition, the introduction of exogenous genes *CePAH1*, *CeDGAT2*, and *CeSAD* into yeast and Arabidopsis significantly increases TAG and total oil content compared to the wild type [6,7,10,11]. However, the complete mechanisms underlying tuber growth and fatty acid synthesis in tiger nut remain to be elucidated. Further investigation is needed to determine the potential existence of other key genes involved in oil synthesis and to understand how the expression levels of the identified genes are regulated.

MicroRNAs are short, endogenous, noncoding RNAs of 18–25 nucleotides (nt) in length found in plants and animals. They regulate the expression levels of their target genes, which are involved in specific biological processes [12,13,14,15]. Numerous miRNAs have been identified in the seeds of oil crops via high-throughput small RNA [16,17,18,19]. For example, 27 miRNAs regulate the gene expression related to seed development and oil accumulation in sea buckthorn [20]. In rapeseed, 32 miRNAs are directly involved in fatty acid synthesis in seeds [16]. Moreover, some miRNAs have been identified as participating directly in organ development or oil synthesis. In rapeseed, miR166f regulates silique length and harvest index [21]. Modified csa-miR159a increases oleic acid content in *Camelina sativa* [22]. In potatoes, the overexpression of miR156 can reduce tuber number and size [14]. Despite the crucial roles of miRNAs in plant growth and oil accumulation, there are currently no reports on miRNAs in tiger nut.

To elucidate the role of miRNAs in tuber development and fatty acid synthesis in tiger nut, we employed GC-MS and small RNA sequencing to obtain dynamic information on fatty acid composition and miRNA changes across five stages of tuber development. Then, bioinformatics tools were used to identify miRNA target genes and their biological annotations, aiming to associate them with tuber traits. Ultimately, this research offers novel insights into the miRNAs and molecular mechanisms underlying tuber development and lipid accumulation in tiger nut.

## 2. Results

### 2.1. Changes in Tiger Nut Tuber Weight, Oil Content and Fatty Acid Composition at Five Development Periods

**Figure 1:**
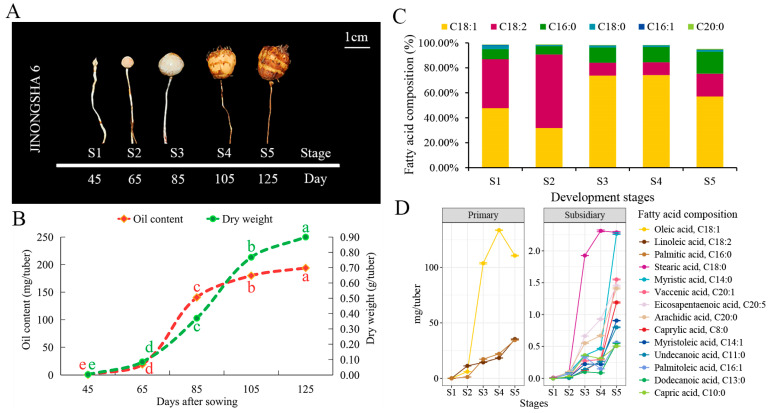
Growth, oil accumulation, and fatty acid composition of tiger nut tubers. (**A**) Five developmental stages of tiger nut tubers. (**B**) Curves of dry matter and oil accumulation. (**C**) Proportion of the six most abundant fatty acids. (**D**) Changes in the accumulation of various fatty acids over the five stages.

In tiger nut cultivar JINONGSHA6, tuber enlargement becomes distinctly observable 45 days after sowing (DAS). As growth progresses, the tuber color transitions from white (S1) to dark brown (S5) (Figure 1A). Tuber weight and oil content both accumulate progressively from S1 to S5 (Figure 1B). A quick increase in the dry weight of the tubers was observed, with the peak growth rate occurring between stages S3 and S4 (85 DAS to 105 DAS), at 0.02 g/day per tuber. The second highest growth rate was observed between stages S2 and S3 (65 DAS to 85 DAS), with an average increase of 0.014 g/day per tuber (Figure 1B). Meanwhile, the fastest rate of oil accumulation occurred between stages S2 and S3 at 6.06 mg/day per tuber, followed by a slow rate of 1.97 mg/day per tuber from stages S3 to S4. These observations indicate that stages S2 to S4 are critical periods for the accumulation of dry matter and oil content in tiger nut.

A total of 14 fatty acids were identified in tiger nut tubers using GC–MS (Table S1). Oleic acid, linoleic acid and palmitic acid were the three most abundant fatty acids at all developmental stages, collectively accounting for over 93% of the total fatty acid content, and are thus considered the primary fatty acids in tiger nut tubers (Figure 1C,D and Figure S1). Furthermore, six unsaturated fatty acids (oleic acid, linoleic acid, palmitoleic acid, myristoleic acid, eicosapentaenoic acid, and vaccenic acid) constituted 75% of the total fatty acid content (Figure S1). Oleic acid comprised over 57% of the total fatty acid content except during the early developmental stages S1 and S2. Meanwhile, the composition ratios of fatty acids changed with developmental stages (Figure 1C). Consistent with the trends in tuber weight and total oil content, the content of most fatty acids accumulated continuously during tuber growth (S4 to S5). Notably, the stages from S2 to S4 were marked by the rapid accumulation of various fatty acids (Figure 1D). For example, the accumulation rates of oleic acid were 4.88 mg/day and 1.49 mg/day during the S2 to S3 and S3 to S4 stages, respectively. In summary, these results indicate that the fatty acids in tiger nut tubers are primarily composed of unsaturated fatty acids, with oleic acid being the most critical component. Additionally, the period from S2 to S4 represents a crucial stage for fatty acid synthesis in the tubers.

### 2.2. Overview of sRNA Sequencing and the Identification of miRNAs

Fifteen small RNA libraries were constructed across five developmental stages to identify the miRNAs involved in material accumulation in tiger nut tubers. A total of 178,164,492 raw reads were obtained from all libraries through high-throughput sequencing, with Q30 values exceeding 95% for each library (Table S2). After the quality control described in the Materials and Methods Section, each library retained between 9,601,609 and 13,391,806 clean reads. Finally, over 94% of the clean reads from each library were mapped to the tiger nut genome. These results demonstrate that the quality of the small RNA sequencing data was sufficient for further analysis.

**Figure 2:**
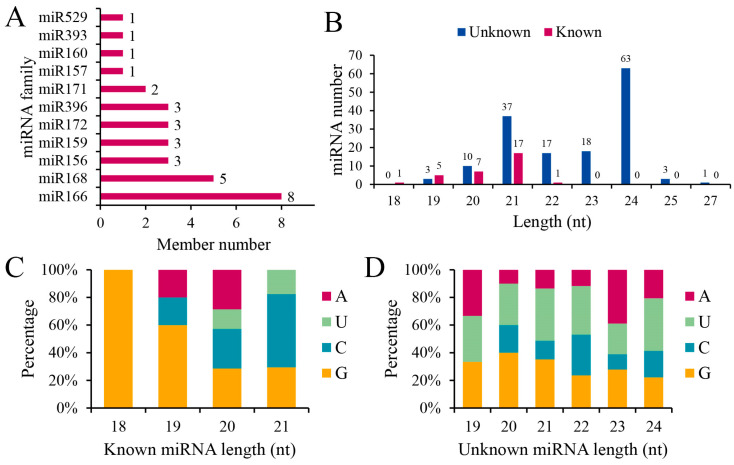
Overview of miRNAs: (**A**) Number of families of known miRNAs; (**B**) Length distribution of miRNAs; (**C**) First nucleotide preference of known miRNAs; (**D**) First nucleotide preference of novel miRNAs.

According to the miRNA analysis pipeline described in the Materials and Methods, we identified 183 mature miRNAs in 15 libraries from five developmental stages of tubers (Table S3). Among these, 31 miRNAs are known plant miRNAs belonging to 11 miRNA families. The miR166 and miR168 families have eight and five members, respectively (Figure 2A). The newly identified miRNA members were named in the form of their ces-miRn-number. In total, 152 novel mature miRNAs, ranging in length from 19 nt to 27 nt, were discovered. Of these, 24.34% have a length of 21 nt, while 41.44% have a length of 24 nt (Figure 2B). In addition, there were differences in the preferential distribution of the first nucleotide between known and novel miRNAs (Figure 2C,D). Known mature miRNAs typically begin with 5′-G and 5′-C, whereas novel mature miRNAs often start with 5′-U and 5′-G.

### 2.3. Differential Expression and Functional Analysis of miRNAs at Different Growth Stages

**Figure 3:**
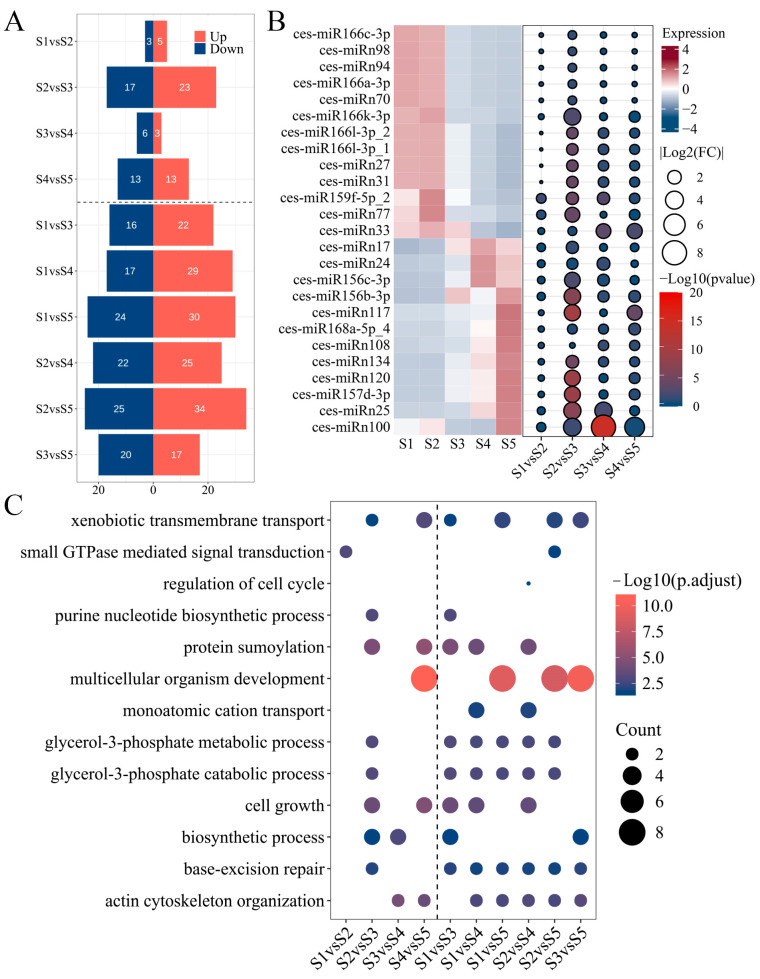
Identification and functional analysis of DEmiRs. (**A**) Number of DEmiRs between any two stages. (**B**) miRNAs with expression changes before and after key stages of metabolite accumulation. (**C**) GO enrichment results of DEmiRs.

Differences in miRNA expression profiles across the developmental stages of tiger nut tubers may be associated with growth and metabolite accumulation. Expression levels were normalized using transcripts per million (TPM), with a threshold for reliable expression set at TPM > 1. The numbers of expressed miRNAs across the five stages were 146, 138, 141, 145, and 139, respectively, resulting in 169 unique miRNAs when combined (Table S4). For differential expression analysis, miRNAs reliably expressed in any two stages were compared, with differentially expressed miRNAs (DEmiRs) defined by an absolute value of fold change (FC) ≥ 1.5 and *p* < 0.05. The analysis revealed that there were fewer DEmiRs between adjacent stages than those between non-adjacent stages (Figure 3A). In comparisons of adjacent developmental stages, the highest number of DEmiRs was observed between S2 and S3, with a total of 40, comprising 23 that were upregulated and 17 that were downregulated. This was followed by 26 DEmiRs between S4 and S5. In contrast, fewer than 10 DEmiRs were identified between S1 and S2, as well as between S3 and S4. Based on miRNA expression levels and differential expression analysis, 25 miRNAs exhibited differential expression between key periods and non-key periods of metabolite accumulation, and their expression level either increased or decreased in accordance with developmental stages (Figure 3B). It is noteworthy that five known miRNAs from the miR166 family showed lower expression levels from S3 to S5 compared to S1 and S2, while two members from the miR156 family displayed an opposite expression pattern to that observed in the miR166 family. Additionally, a novel miRNA, ces-miRn100, showed a very reduced expression (TPM < 1) in S3 and S4, but its expression level in S5 was higher than in S1 and S2, suggesting a potential close association with tuber metabolite synthesis.

The target genes of miRNAs were predicted to assess their potential functions, resulting in the identification of 618 target genes, 102 of which were annotated as transcription factors (Table S5). Gene ontology (GO) and KEGG enrichment analyses were conducted to elucidate the biological processes in which target genes of DemiRs were involved at various growth stages. GO annotations indicated that significantly enriched biological processes were related to cell growth and glycerol phosphate metabolism (Figure 3C). Remarkably, most of the previously mentioned target genes of 25 DEmiRs were involved in these pathways. For instance, pathways associated with glycerol-3-phosphate metabolism were enriched during both early and later developmental stages. In these pathways, two glycerol-3-phosphate dehydrogenases (GPDH) were regulated by ces-miR156b-3p, which showed low expression levels during early development but consistently high expression from stages S3 to S5 (Figure 3B). Processes such as multicellular organism development, actin cytoskeleton organization, and base-excision repair were significantly enriched at key early and late developmental stages (Figure 3C). These processes involve eight APETALA2/ethylene-responsive (AP2/ERF) transcription factors, two suppressors of cAMP receptor (SCAR-LIKE) proteins, and two endonuclease III enzymes. Specifically, ces-miR172b-3p, ces-miR172d-3p, and ces-miRn2 target eight *AP2*, exhibiting high expression only at stage S5 (Table S4). The two SCAR-LIKE proteins are regulated by ces-miRn108, which shows progressively increased expression levels during tuber growth. While no expression differences are observed from stages S1 to S3, distinct differences emerge at stages S4 and S5 compared to earlier stages (Figure 3B). Endonuclease III is targeted by five miRNAs: ces-miRn27, ces-miRn31, ces-miR166l-3p_1, miR166l-3p_2, and miR166k-3p. These miRNAs maintain high expression levels at stages S1 and S2, but their expression is reduced to one-fourth of their previous abundance after S3 (Figure 3B and Table S4). The KEGG enrichment results were close to the GO analysis (Figure S2). Differing from these results, the pathway “starch and sucrose metabolism” was significantly enriched according to the KEGG analysis. The highly expressed ces-miR168a-5p_4 in S4 and S5 regulated three isoamylase 1 enzymes in this pathway (Figure 3B). Additionally, the folate biosynthesis pathway was only not significantly enriched in the S1vsS2 and S4vsS5 comparisons. This pathway involved three aminodeoxychorismate synthases which were targeted by ces-miRn25. This novel miRNA, like ces-miR168a-5p_4, showed a gradual increase in expression during tuber growth (Figure 3B). In conclusion, the miRNA expression profiles at various growth stages corresponded to the curves of tuber growth and metabolites accumulation, and their target genes were mainly involved in cell growth, glycerophosphate, and sugar synthesis.

### 2.4. miRNA–mRNA Regulatory Network Involved in Metabolite Sythesis and Development Progress in Tiger Nut Tubers

**Figure 4:**
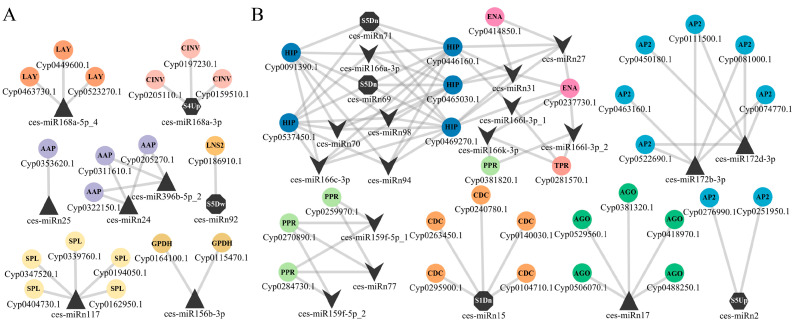
Hypothetical miRNA–mRNA interaction networks. (**A**) Metabolite synthesis of tubers. (**B**) Growth and development of tubers.

To further understand the regulatory mechanisms of miRNAs on tiger nut tuber growth and metabolite synthesis, a miRNA–mRNA regulatory network was constructed using DEmiRs information and function annotations of target genes in Cytoscape v3.10.2 [23]. A total of eight miRNAs were identified to be associated with the synthesis of fatty acids, amino acids, and carbohydrates (Figure 4A), and these miRNAs generally exhibited higher expression levels starting from S3 (Figure 3B and Table S4). In addition to ces-miR156b-3p-*Cyp0115470.1/Cyp0164100.1* (GPDH), the interaction pair related to fatty acid synthesis included ces-miRn92-*Cyp0186910.1* [Lipin/Ned1/Smp2 (LNS2)] and ces-miRn117-*Cyp0194050.1*/*Cyp0339760.1*/*Cyp0347520.1*/*Cyp0404730.1* [squamosa promoter-binding-like protein (SPL)]. Notably, the expression level of ces-miRn92 decreased only at S5 (Table S4). The amino acid metabolism involved two miRNA–mRNA interaction pairs: ces-miR396b-5p_2/ces-miRn24-*Cyp0205270.1*/*Cyp0311610.1*/*Cyp0322150.1* [amino acid permeases (AAP)] and ces-miRn25-*Cyp0353620.1* [amino acid permeases (AAP)]. Two miR168as are involved in carbohydrate metabolism. miR168a-5p_4 interacted with three isoamylase 1 genes: *Cyp0449600.1*, *Cyp0463730.1*, and *Cyp0523270.1*. miR168a-3p regulated three alkaline neutral invertase CINV2-like proteins (CINV): *Cyp0159510.1*, *Cyp0197230.1*, and *Cyp0205110.1*. This miRNA only exhibited higher expression levels at S4 (Table S4).

On the other hand, the miRNA–mRNA regulatory network related to growth and development processes included 22 miRNAs (Figure 4B); of these, 13 miRNAs exhibited lower expression levels after S3 (including S3) compared to earlier developmental stages (S1 and S2) (Figure 3B and Table S4). The largest module consisted of five miR166 family members (ces-miR166a-3p, ces-miR166c-3p, ces-miR166k-3p, ces-miR166l-3p_1, and ces-miR166l-3p_2) and seven novel miRNAs (ces-miRn27, ces-miRn31, ces-miRn69, ces-miRn70, ces-miRn71, ces-miRn94, and ces-miRn98); their target genes included five HD-ZIP (*Cyp0091390.1*/*Cyp0446160.1*/*Cyp0465030.1*/*Cyp0469270.1*/*Cyp0537450.1*), two endonuclease III (*Cyp0237730.1* and *Cyp0414850.1*), one pentatricopeptide repeat-containing protein/PPR (*Cyp0381820.1*), and one tetratricopeptide repeat protein/TPR (*Cyp0281570.1*). The remaining miRNA–mRNA interactions only involved a single gene family. Among these, ces-miR159f-5p_1, ces-miR159f-5p_2, and ces-miRn77 collectively acted on three PPR genes: *Cyp0259970.1*, *Cyp0270890.1*, and *Cyp0284730.1*. Meanwhile, ces-miR172b-3p and ces-miR172d-3p regulated six *AP2* genes: *Cyp0074770.1*, *Cyp0081000.1*, *Cyp0111500.1*, *Cyp0450180.1*, *Cyp0463160.1*, and *Cyp0522690.1*. Notably, the expression levels of these two miRNAs were higher in S4 compared to the first three stages (S1, S2, and S3) (Table S4). In another interaction pair, ces-miRn2-*Cyp0251950.1*/*Cyp0276990.1*, ces-miRn2 also acted on two AP2 genes and its expression level in S5 was more than twice as high as that in the other four stages (Table S4). Ces-miRn17, which was highly expressed in the later stages of development (Figure 3B), regulated five argonaute/AGO proteins (*Cyp0381320.1*/*Cyp0418970.1*/*Cyp0488250.1*/*Cyp0506070.1*/*Cyp0529560.1*). Ces-miRn15 participated in cell division by regulating five cell division cycle proteins/CDC (*Cyp0104710.1*/*Cyp0140030.1*/*Cyp0240780.1*/*Cyp0263450.1*/*Cyp0295900.1*), and its expression level was only low in S1 (Table S4).

### 2.5. Quantitative Real-Time PCR (qRT-PCR) Validation of miRNA and Their Target Genes

**Figure 5:**
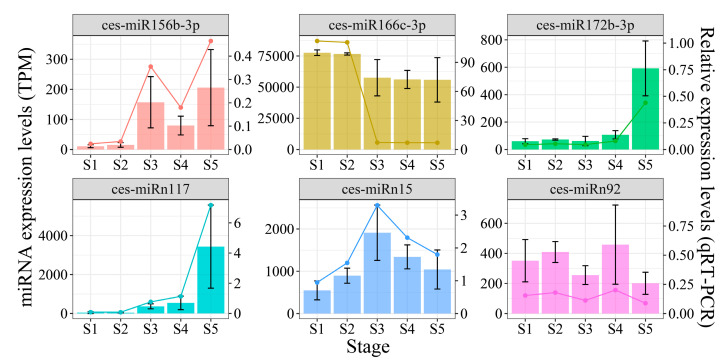
qRT-PCR validation of the six DEmiRs at five development stages (bar plot shows miRNA expression from small RNA sequencing and line plot shows relative expression from qRT-PCR).

To assess the validity of small RNA sequencing, six miRNAs were randomly selected from two regulatory networks, and qRT-PCR was conducted on samples from five developmental stages of tiger nut tubers, with *CeU6* serving as the housekeeping gene. The results showed that the TPM values and relative expression levels of these six miRNAs displayed consistent trends across all five developmental stages (Figure 5). These findings confirm the robustness of high-throughput sequencing.

**Figure 6:**
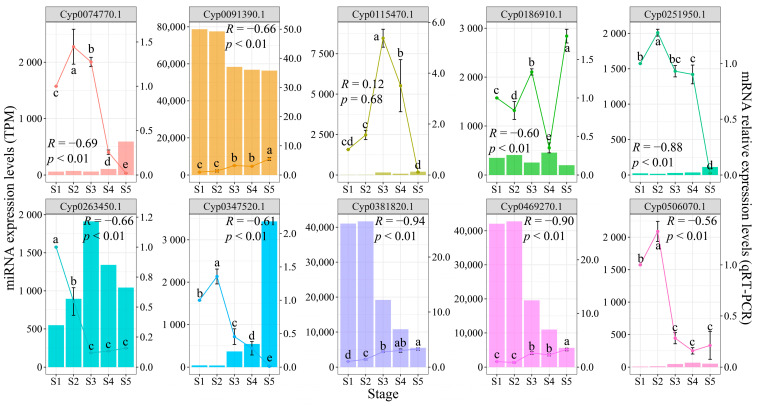
Expression analysis between target genes and their corresponding miRNA. The line plots represent the relative expression levels of mRNAs, with data shown as means ± standard deviation from three independent experiments. Different lowercase letters indicate significant differences (*p* < 0.05, Duncan’s multiple range tests). The bar charts show miRNA expression levels, and the correlations between mRNAs and miRNAs were analyzed using Pearson’s correlation coefficient (*p* < 0.05 indicates significant correlation and *p* < 0.01 indicates highly significant correlation). For target genes regulated by multiple miRNAs, only the correlation with the lowest *p* value is shown in the figure.

Furthermore, to validate the miRNA–mRNA regulatory relationships, 10 target genes were randomly selected from the predicted interaction network (Figure 4). The relative expression levels of these mRNAs varied according to tuber growth (Figure 6), with notable differences observed between critical periods of metabolite accumulation or development (one-way analysis of variance followed by Duncan’s multiple range tests, *p* < 0.05). For instance, *Cyp0074770.1* showed significantly higher expression during S2 and S3 compared to other stages, while being barely detectable in later developmental stages. The expression analysis revealed that the majority of miRNAs exhibited highly significant (Pearson’s correlation, *p* < 0.01) or significant (*p* < 0.05) negative correlations with their target genes (Figure 6 and Table S7). However, three mRNA–miRNA pairs showed no significant correlations: *Cyp0469270.1* and *Cyp0091390.1* with ces-miRn69 and ces-miRn71, and *Cyp0115470.1* with ces-miR156b-3p. Nevertheless, both *Cyp0469270.1* and *Cyp0091390.1* were identified as potential targets of multiple miRNAs (Figure 4), and all these relationships demonstrated highly significant negative correlations (Table S7).

## 3. Discussion

Tiger nut is a plant known for its ability to accumulate substantial amounts of oil in its tuber [24], making it essential for understanding the patterns and mechanisms of oil production. This study utilized GC–MS to determine the accumulation and changes in the oil content and fatty acid composition of tubers at five developmental stages. Consistent with previous findings, the tubers are rich in various unsaturated fatty acids, with oleic acid constituting more than 50% of the total oil content, and thus being the predominant component [6,9]. Moreover, both dry matter weight and total oil content increase rapidly during the mid-developmental stages and remain elevated for over 40 days (Figure 1B) [6,8,9]. Among the 14 detected fatty acids, this trend is particularly evident for oleic acid [6]. Notably, in the later developmental stages, the levels of major fatty acids such as oleic acid, linoleic acid, and palmitic acid decrease, while those of minor fatty acids increase (Figure 1C,D and Figure S1). This may reflect shifts in the synthesis and consumption of fatty acid components. Overall, our results suggest that 65 DAS to 105 DAS (from 35 to 75 days after tuber formation) are critical for metabolite accumulation in tiger nut tubers.

miRNAs have been demonstrated to influence the oil content and composition of plant seeds and are crucial in potato tuber development [12,14,15,25]. In this study, we identified 183 miRNAs using high-throughput sequencing and mapped their expression profiles (Tables S3 and S4). Among these, miR166, miR168, and miR156 were identified in 11 miRNA families within the tubers (Figure 2A). These miRNAs are also found in oil-rich plants such as rapeseed [16], sesame [26], and peony [17], indicating their conserved nature. Nonetheless, a significant number of unidentified miRNA types were observed in tiger nut (Table S3). Some miRNAs known to be involved in plant fatty acid and oil production, such as miR169 and miR390, were not detected, which may be due to interspecies differences [16,26]. These results underscore the need for small RNA studies across various tissues and cultivars to enhance our understanding of miRNA types and functions in diverse plants.

The analysis of differential miRNA expression and the functions of their target genes at various developmental stages in plants provide insights into how miRNAs mediate biological functions [17,18]. For instance, the rapid oil accumulation stage in sesame seeds coincides with the most significant changes in miRNA expression, with these miRNAs’ target genes being involved in numerous biological processes related to lipid synthesis [26]. Similarly, in tiger nut tubers, the expression changes of many miRNAs across five developmental stages closely align with the rate of metabolite accumulation (Figure 3B). During the stages of the most rapid synthesis of dry matter, total oil, and fatty acids, there is an increased number of DEmiRs (Figure 3A). These miRNAs directly target genes involved in the glycerol-3-phosphate catabolic process and cell growth (Figure 3C and Table S5). The ces-miR156-3p directly targets two *GPDH* genes (Figure 4A), with its expression significantly increasing at 85 DAS and remaining at high levels thereafter (Figure 3B). As a key enzyme in the Kennedy pathway, GPDH catalyzes the synthesis of glycerol-3-phosphate. In soybeans, *GmGPDH* overexpression can directly increase diacylglycerol content and promote oleic acid synthesis, leading to simultaneous increases in both soybean oil and oleic acid content [27]. Unexpectedly, although the *GPDH* gene *Cyp0115470.1* shows higher relative expression levels during S3 and S4 compared to other stages, it exhibits only a weak positive correlation with ces-miR156-3p expression (Figure 6). These findings suggest that the interaction between *GPDH*, a component of the oil biosynthesis pathway in tiger nut tubers, and ces-miR156-3p might be more complex than anticipated. Ces-miRn117, which shares a similar expression pattern with ces-miR156-3p, targets five *SPL* genes (Figure 4A). In rapeseed, miR156 interacts with *SPL2/10* and *SPL10/11* to regulate oil production and seed maturation, respectively [28,29]. *StSPL9* is involved in potato tuber formation through tis regulation of the expression levels of miR172. *Cyp0347520.1*, identified as an *SPL* gene, exhibited high expression levels during the S2 stage and was progressively suppressed as the tuber matured (Figure 6). This expression pattern suggests that it might play a role in regulating oil accumulation during the rapid oil biosynthesis phase (Figure 6). Ces-miR172b-3p and ces-miR172d-3p are key modules in tuber growth, targeting six *AP2* genes (Figure 4B). Two reports indicate that *AP2* genes not only control the size and number of Arabidopsis seeds but also influence dry mass and oil accumulation [30,31]. *Cyp0074770.1*, an *AP2* gene, as a target gene of two Ces-miR172 family members, was found to be transcriptionally repressed (Figure 6). This gene exhibits higher expression levels during the S2 to S3 stages compared to other developmental stages, with minimal expression detected in the later developmental phases. Furthermore, the miR172 family has been identified as a key factor mediating fat formation in oilseed crop seeds [16,17,26]. Thus, ces-miRn117-*SPL* and ces-miR172-*AP2* may interact to jointly regulate the oil synthesis and development of tuber.

In addition, ces-miR168a-5p_4, which regulates four genes encoding isoamylase, involved in starch accumulation in seeds or tubers [32,33], exhibited a rapid increase in expression to twice its previous level at 85 DAS and further increased as the tubers matured (Figure 3B and Table S4). These results suggest that miR168a-5p_4 is involved in the tuber starch synthesis pathway. Notably, several studies have demonstrated that DGAT [10,11] and SAD [7] play a crucial role in oil accumulation in tiger nut tubers. However, the miRNAs identified in this study did not act on DGAT or SAD, highlighting the complexity of oil production mechanisms in tiger nut tubers. Thus, future research should conduct transcriptomic and other omics studies across different varieties of tiger nut, particularly among those with significant differences in oil content, to elucidate the regulatory mechanisms of miRNAs in tuber oil accumulation.

miR166 family members are highly conserved and commonly found in oilseed crops, representing a significant group of miRNAs [17,19]. The miR166-*HD-ZIP* module is a key regulator of plant organ growth [13,15,21]. In this study, miR166, one of the most abundant conserved miRNA families, showed that the expression levels of five members gradually decreased during development or were repressed after 85 DAS, maintaining their expression at less than 1.3-fold of the previous level or becoming undetectable (Figure 3B and Table S4). Two HD-ZIP family target genes, *Cyp0091390.1* and *Cyp0469270.1*, which were negatively regulated by miR166, exhibited progressively increased expression levels during tuber maturation. The relative expression levels of these genes at the S5 stage were more than three-fold higher compared to those at the S1 stage (Figure 6 and Table S7). Remarkably, ces-miRn17 not only regulates five AGO proteins but also displays an expression pattern that is opposite to that of the five miR166 members (Figure 3B and Figure 4B and Table S4). Moreover, one of the *AGO* genes, *Cyp0506070.1*, exhibited high expression levels exclusively during the S1 and S2 stages (Figure 6). Previous studies have demonstrated that *AtAGO1* regulates vegetative organ development by activating miR166, which in turn suppresses *HD-ZIP* gene expression [15]. Hence, the ces-miRn17-*AGO*-ces-miR166-*HD-ZIP* module may form a similar regulatory network to influence the growth and development of tiger nut tubers. Meanwhile, these miR166 members, together with ces-miRn27, ces-miRn31, and others, form an extensive regulatory network targeting *HD-ZIP*, *endonuclease III*, *PPR*, and *TPR* genes (Figure 4B). The loss of the *AtTPR* in Arabidopsis can cause abnormal cell proliferation [34]. In addition, the *CDC48* gene directly regulates the cell division process [35,36]. Ces-miR15 targets five *CDC48* genes, with its expression level increasing twofold after 65 DAS and remaining elevated until tuber maturation (Figure 4B and Table S4). In contrast, the expression of the *CDC48* gene (*Cyp0263450.1*) showed an inverse pattern to Ces-miR15, with its expression level sharply declining after S1 and becoming barely detectable in subsequent stages (Figure 6). Therefore, it can be inferred that cell division in tiger nut tubers primarily occurs during the first 35 days of tuber formation, with ces-miR15 playing a crucial role. In conclusion, the results demonstrate that miRNAs are closely associated with oil accumulation, fatty acid composition, and growth during the developmental stages of tiger nut tubers. However, further validation is required to elucidate how miRNA–mRNA interactions influence these traits.

## 4. Materials and Methods

### 4.1. Plant Materials

The tiger nut cultivar, JINONGSHA6, was planted in the experimental field of Jilin Agricultural University (Jilin Province, China). In 2022, tiger nut tubers began to form 30 DAS and reached maturity at 125 DAS. Consequently, five developmental stages were selected for this study: 45 DAS (S1), 65 DAS (S2), 85 DAS (S3), 105 DAS (S4), and 125 DAS (S5). Uniform-sized tubers were obtained from three plants exhibiting similar growth, washed with distilled water to remove soil, and dried with absorbent paper. They were then immediately frozen in liquid nitrogen and subsequently stored in a −80 °C freezer for further analysis

### 4.2. Analysis of Oil Content and Fatty Acid Composition

A total of 200 mg of tiger nut tuber powder was mixed with 5 mL of 5% hydrochloric acid methanol solution, 10 mL of dichloromethane, and 15 mL of methanol. The mixture was heated in an 80 °C water bath for 1 h and then cooled to 25 °C. The solution was adjusted to a final volume of 20 mL with methanol to obtain the test solution. The sample solution was prepared by mixing 1 mL of the test solution with an internal standard solution (methyl nonadecanoate) and 5% aqueous ammonia. The oil content and fatty acid profile of the samples were determined by GC–MS using a Trace 1310 ISQ (Thermo Fisher Scientific, Waltham, MA, USA). Separation was performed on a TG-5MS capillary column (30 m × 0.25 mm × 0.25 μm) with helium as the carrier gas at a flow rate of 1.2 mL/min. A split injection mode with a split ratio of 1:50 was used, and the injector temperature was set to 290 °C. The oven temperature was programmed to start at 80 °C for 1 min, ramp to 200 °C at a rate of 10 °C/min, hold at 250 °C for 5 min, then increase to 270 °C at a rate of 2 °C/min, and hold at 270 °C for 3 min. The temperatures of the transfer line and ion source were set to 280 °C. The mass spectrometry scanning range was 30–400 amu. All experiments were performed in triplicate. A statistical analysis of tuber traits was conducted using the R v4.3.1 (https://www.r-project.org/ (accessed on 6 October 2024)). For comparisons among multiple groups, one-way analysis of variance (ANOVA) and Duncan’s multiple range test were conducted using the R package agricolae [37].

### 4.3. RNA Isolation and Small RNA Sequencing

Total RNA from 15 samples at five tuber developmental stages was extracted using the RN40-EASYspin reagent (Aidlab, Beijing, China) according to the manufacturer’s instructions. The quality and quantity of the total RNA from each sample were analyzed using a NanoDrop 2000 spectrophotometer (Thermo Fisher Scientific, Waltham, MA, USA). Small RNA was separated from high-quality RNA and used to construct small RNA libraries with the NEBNext Ultra Small RNA Library Prep Kit for Illumina (NEB, Ipswich, MA, USA). Sequencing was performed on the Illumina NovaSeq 6000 platform in single-end 50 bp (Biomarker Technology, Beijing, China).

Raw reads were processed by removing the adaptor sequence using Trimmomatic v0.39 with the “SE” module [38]. Low-quality reads, poly-A tails, shorter than 18bp, and longer than 34 bp were filtered using Cutadapt v4.1 [39] with the parameter setting “-a A{20} -m 18 -M 34”. Clean reads from all samples were combined to identify known and novel miRNAs. Combined reads were converted into unique tags using the FASTX-Toolkit 0.0.14 (https://www.encodeproject.org/software/fastx_toolkit/ (accessed on 6 October 2024)). Ribosomal RNA (rRNA), transfer RNA (tRNA), small nuclear RNA (snRNA), small nucleolar RNA (snoRNA), and repeat sequences were filtered by aligning unique tags to the Rfam database [40] and Repbase database [41]. Known miRNAs in tiger nut were identified based on known plant miRNAs in the miRbase database v22 [42]. To accurately identify novel miRNAs, the plant-specific miRNA analysis tool miRDeep-P2 [43] was used to identify novel miRNAs with the minor modification [44]. The chromosome-level genome assembly sequence of tiger nut (unpublished) was available to detect novel miRNA. A total of 68,504 coding sequences from the gene annotation were applied to predict miRNA targets using psRNATarget [45]. The eggNOG-mapper v2 [46] and plantTFDB v5.0 [47] were used for the functional annotation of genes and identification of transcription factors based on gene protein sequences.

### 4.4. Differential Expression Analysis of miRNAs

After the identification of known and novel miRNAs, the expression of miRNAs in each sample was quantified by aligning clean reads to the final set of miRNA sequences using Bowtie v1.3.1 with default parameters [48]. The read counts for each miRNA in each sample were generated using SAMtools [49]. The expression level of miRNA was normalized to TPM. The differential expression of miRNAs was analyzed based on read counts using DESeq2 across different developmental stages [50,51]. Differentially expressed miRNAs were identified using an absolute value of FC ≥ 1.5 and a *p*-value < 0.05. GO and KEGG enrichment analyses were performed for the target genes of differentially expressed miRNAs between different stages using the clusterProfiler 4.0 [52]. All figures were plotted using ggplot2.

### 4.5. Expression Analysis of miRNA and Their Target Genes Using qRT-PCR

The qRT-PCR analysis of miRNAs and their target genes was performed using a QuantStudio^TM^ 5 Real-Time PCR System (Thermo Fisher Scientific, Waltham, MA, USA). For miRNAs, residual total RNA from small RNA library construction was reverse-transcribed to cDNA using TransScript miRNA First-Strand cDNA Synthesis SuperMix (AT351, TransGen, Beijing, China) according to the manufacturer’s protocol. qRT-PCR was conducted using TransStart Top Green qPCR SuperMix (AQ132-11, TransGen, Beijing, China), with *CeU6* as the internal reference control. The qRT-PCR cycling conditions were as follows: initial denaturation at 95 °C for 2 min, followed by 45 cycles of 95 °C for 15 s and 60 °C for 30 s. For mRNA analysis, total RNA was reverse-transcribed using StarScript III RT Kit (A232, GeneStar, Beijing, China), with *CeUCE2* as the reference gene [53]. Subsequent steps were identical to those for miRNA. All samples were subjected to three technical replicates. Relative expression levels were calculated using the relative quantification method (2^−ΔΔCt^) [54]. The primers used for qRT-PCR are listed in Supplementary Table S6. Additionally, Pearson correlation coefficients between the relative expression of mRNAs and the TPM of their corresponding miRNA were calculated using the R package corrplot 0.92.

## 5. Conclusions

This study reveals that metabolite accumulation, predominantly oleic acid, in C. esculentus tubers primarily occurs from 65 DAS to 105 DAS. Small RNA sequencing across five developmental stages identified 183 miRNAs, comprising 31 known miRNAs from 11 families and 152 novel miRNAs. Significant differences in miRNA expression were observed during key periods of metabolite accumulation. The target genes of these miRNAs are significantly enriched in glycerate 3-phosphate synthesis and cell growth processes, and 13 related regulatory networks were identified. For instance, ces-miR156b-3p-*GDPH* and ces-miRn117-*SPL* are associated with total oil content and oleic acid synthesis, while ces-miRn17-*AGO*-ces-miR166-*HD-ZIP* and ces-miR15-*CDC48* regulate tuber growth and development. miRNAs primarily participate in biological processes by suppressing the expression of their target genes. These findings provide insights into the key miRNAs related to metabolite accumulation and their regulatory networks with target genes in crops where tubers are the main economic organ, enhancing our understanding of the developmental and oil production mechanisms in tiger nut tubers.

## Data Availability

All small RNA sequencing data have been deposited at the NGDC website (https://ngdc.cncb.ac.cn/), and the bioproject number is PRJCA031104.

## Supplementary Materials

The following supporting information can be downloaded at: https://www.mdpi.com/article/10.3390/plants13233305/s1, Figure S1: Content of various fatty acids of tiger nut tubers at five development stages; Figure S2: KEGG enrichment analysis of differentially expressed miRNAs, Table S1: Fatty acid composition of tiger nut tubers at different developmental stages; Table S2: Summary of small RNA sequencing quality; Table S3: Basic information of 183 identified miRNAs; Table S4: Expression levels of miRNAs at different developmental stages; Table S5: Target genes of miRNAs and their annotation information; Table S6: Primers for qRT-PCR analysis of miRNAs and their target genes; Table S7: Correlation between miRNAs and their target genes.
