# Supplementary material for: Identification of miRNAs Involved in Lipid Metabolism and Tuber Development in Cyperus esculentus L
Source: Plants (Basel). 2024 Nov 25;13(23):3305. doi: 10.3390/plants13233305 (PMC11644708; doi:10.3390/plants13233305)
Supplement: Supplementary file 1 [file plants-13-03305-s001.zip › Figure S1 Content of various fatty acids of tiger nut tubers at five development stages.pdf]

Fatty acid	Fatty acid content in five developmental stages (mg/tuber)				
	S1	S2	S3	S4	S5
Oleic acid, C18:1	0.2038±0.0013 e	6.1075±0.0165 d	103.7271±1.0930 c	133.5946±0.3644 a	110.6531±0.6522 b
Linoleic acid, C18:2	0.1675±0.0011 e	11.3300±0.0543 d	14.5186±0.1389 c	18.3143±0.2205 b	35.3898±0.2862 a
Palmitic acid, C16:0	0.0349±0.0002 e	1.3049±0.0066 d	17.2307±0.0584 c	22.0601±0.2864 b	34.4528±0.2174 a
Stearic acid, C18:0	0.0123±0.0000 d	0.0957±0.0010 c	1.9268±0.0020 b	2.3138±0.0244 a	2.2891±0.0266 a
Palmitoleic acid, C16:1	0.0021±0.0000 e	0.0692±0.0009 d	0.3359±0.0025 b	0.1503±0.0016 c	0.5611±0.0066 a
Myristic acid, C14:0	0.0017±0.0000 e	0.0971±0.0010 d	0.3578±0.0031 c	0.4655±0.0071 b	2.2700±0.0255 a
Vaccenic acid, C20:1	0.0012±0.0000 e	0.0163±0.0000 d	0.2738±0.0010 c	0.2952±0.0020 b	1.5504±0.0129 a
Arachidic acid, C20:0	0.0011±0.0000 e	0.1041±0.0009 d	0.5508±0.0063 c	0.6684±0.0075 b	1.4100±0.0149 a
Dodecanoic acid, C13:0	0.0006±0.0000 e	0.0161±0.0001 d	0.1015±0.0006 b	0.0871±0.0010 c	0.5463±0.0009 a
Myristoleic acid, C14:1	0.0006±0.0000 d	0.0174±0.0001 c	0.2246±0.0023 b	0.2240±0.0018 b	0.9049±0.0073 a
Eicosapentaenoic acid, C20:5	0.0004±0.0000 e	0.0258±0.0001 d	0.6622±0.0040 c	0.9286±0.0113 b	1.4543±0.0146 a
Caprylic acid, C8:0	0.0003±0.0000 e	0.0282±0.0003 d	0.3607±0.0016 b	0.3073±0.0042 c	0.5043±0.0064 a
Capric acid, C10:0	0.0003±0.0000 d	0.0112±0.0001 d	0.1303±0.0012 c	0.2706±0.0014 b	1.1893±0.0157 a
Undecanoic acid, C11:0	0.0003±0.0000 d	0.0061±0.0000 d	0.1402±0.0016 c	0.2687±0.0002 b	0.7990±0.0119 a
