# Supplementary figures and images for: Identification of miRNAs Involved in Lipid Metabolism and Tuber Development in Cyperus esculentus L
Source: Plants (Basel). 2024 Nov 25;13(23):3305. doi: 10.3390/plants13233305 (PMC11644708; doi:10.3390/plants13233305)

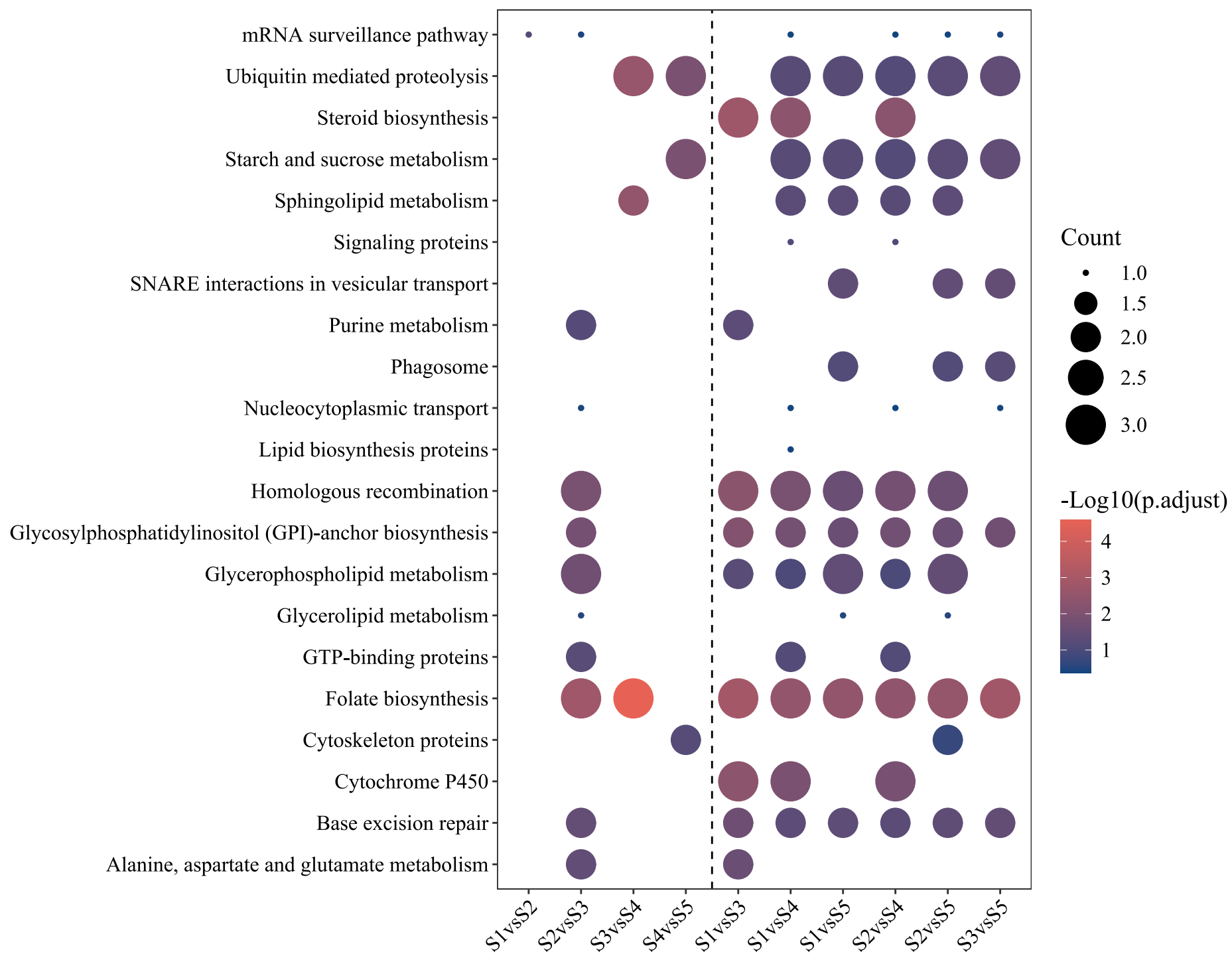

Supplement: Supplementary file 1 [file plants-13-03305-s001.zip › Figure S2 KEGG enrichment analysis of differentially expressed miRNAs.pdf]
